# Identifying asymptomatic *Leishmania* infections in non-endemic villages in Gedaref state, Sudan

**DOI:** 10.1186/s13104-019-4608-2

**Published:** 2019-09-11

**Authors:** Nouh Saad Mohamed, Hussam A. Osman, Mohamed S. Muneer, Abdallah M. Samy, Ayman Ahmed, Anwar O. Mohammed, Emmanuel Edwar Siddig, Muzamil M. Abdel Hamid, Mohamed Siralkhatim Ali, Rihab A. Omer, Arwa H. Elaagip

**Affiliations:** 1 Department of Parasitology and Medical Entomology, Faculty of Medical Laboratory Sciences, University of Sinner, 11111 Khartoum, Sudan; 2 Department of Parasitology and Medical Entomology, Faculty of Medical Laboratory Sciences, Nile University, Khartoum, Sudan; 3 Department of Molecular Biology, Institute of Zoology, University of Hohenheim, Stuttgart, Germany; 4 Biomedical Research Laboratory, Ahfad University for Women, Omdurman, Sudan; 5 Department of Neurology, Mayo Clinic, Jacksonville, FL USA; 6 Department of Radiology, Mayo Clinic, Jacksonville, FL USA; 7 Department of Internal Medicine, Faculty of Medicine, University of Khartoum, Khartoum, Sudan; 8 Entomology Department, Faculty of Science, Ain Shams University, Abbassia, Cairo 11566 Egypt; 9 Department of Parasitology and Medical Entomology, Institute of Endemic Diseases, University of Khartoum, Khartoum, Sudan; 10 Malaria Control Program and Vector Control, Gedaref Ministry of Health, Gedaref, Sudan; 11 Department of Basic Medical Sciences, Faculty of Medical Laboratory Sciences, University of Khartoum, Khartoum, Sudan; 12 Mycetoma Research Center, University of Khartoum, Khartoum, Sudan; 13 School of Medicine, Nile College, Khartoum, Sudan; 14 Department of Molecular Biology, National University Research Institute, National University, Khartoum, Sudan; 15 Faculty of Medicine, Neelain University, Khartoum, Sudan; 16 Department of Molecular Biology, Institute of Parasitology, University of Leipzig, Leipzig, Germany; 17 Department of Parasitology and Medical Entomology, Faculty of Medical Laboratory Sciences, University of Khartoum, Khartoum, Sudan

**Keywords:** Visceral leishmaniasis, Asymptomatic infection, Non-endemic villages, Gedaref state, Sudan

## Abstract

**Objectives:**

Infection with the causative agent of visceral leishmaniasis (VL) may be either symptomatic or asymptomatic. In this study we aimed at investigating the prevalence of asymptomatic infections of *leishmania* in non-endemic villages in Gedaref state, Sudan. A descriptive cross-sectional study conducted during September and October 2014. Blood samples were collected for serological and molecular analysis. Sticky-traps, knockdown spray and CDC-miniature light traps were used for the collection of sandflies.

**Results:**

Ninety-Five participants were included; 52 from Abukishma, 15 Algadamblia Tirfa, 25 Abualnaja and 3 were from Algadamblia Aljabal. Females constituted 56 (58.9%) of the study participants while males were 39 (41.1%). The most frequent age group was > 40-years (54.7%). *Balanites/Acacia* trees were the most planted tree inside the houses; 78 (82.1%). Also, 85 (89.5%) of the participants breed animals inside the house. DAT test revealed 5 positive participants (5.2%). 4/5 DAT positive were past VL infection. PCR detected 35 (36.8%) positive participants. A total of 31/35 was considered asymptomatic infections based on PCR. Households planted *Balanites*/*Acacia* trees or breed domestic animals were found in high percentages with *L. donovani* PCR positive participants (60.1%, 91.4%). No statistically significant was found for VL associated risk factors and VL asymptomatic participants.

## Introduction

Visceral leishmaniasis (VL), also known as new world leishmaniasis or Kala-azar, is a neglected tropical disease caused by protozoan parasites of the *Leishmania donovani* complex. The parasites are transmitted through bites of infected *phlebotomine* sandfly vectors [1]. The disease is endemic in southern Europe, Latin America, Asia, and Africa with an estimated burden of 875; 3668; 45,119; and 8569 cases per annum, respectively [1]. Individuals with VL are clinically defined as symptomatic when there is a prolonged, persistent fever (i.e., longer than 2 weeks) and wasting with progressive spleen enlargement [2, 3]. However, subclinical infections can as well advance to apparent illness [4]. VL has long been endemic in Sudan, and it remains a significant public health problem most notably in areas around Sudan-Ethiopia borders and White Nile State in central-eastern and western Sudan. [5–7]. These areas are characterized by high incidence, morbidity, and mortality [8]. In Sudan, only *Phlebotomus orientalis* has been implicated as vector to transmit VL despite the presence of other circulating sandfly species; *P. papatasi*, *P. saevus, P. rodhaini, Sergentomyia clydei, S. antennata, S. sckwetzi, S. Africana*, and *S. squamipleuris* [9]. In eastern Sudan, there are consistently high rates of infection, with about 16% death rate attributed to VL [5]. However, in most endemic regions, the classical form of the disease is only manifested in about 20% of the infected population, while the majority remain asymptomatic and may progress to symptomatic state or resolve the infection [10, 11]. These asymptomatic populations remain a potential reservoir for maintaining transmission cycles in endemic areas, and as well as re-introducing infections in non-endemic regions [12]. Although, investigation and analysis of VL risk factors have been conducted in various VL foci [10, 12–16]. This study, therefore aimed at investigating the prevalence of asymptomatic infections in non-endemic villages in Gedaref state, Sudan.

## Main text

### Materials and methods

#### Study design and study area characteristics

**Fig. 1:**
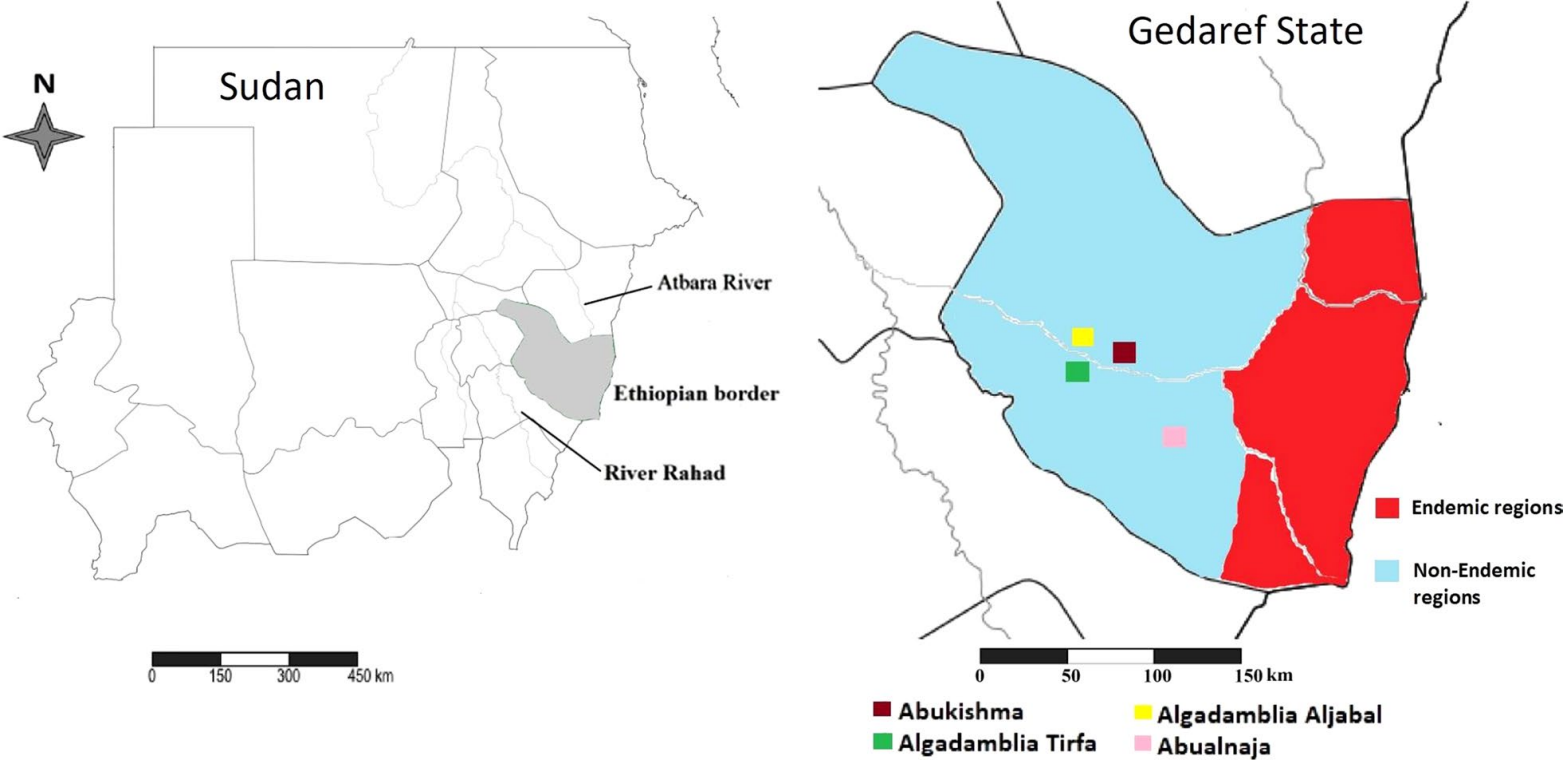
Map of Gedaref State showing the study villages in Gedaref state, Sudan

A descriptive cross-sectional hospital-based study was conducted in Gedaref State, eastern Sudan between September and October 2014. Four villages were selected based on the reports of the presence of 4 new VL cases from the Leishmaniasis Control Program, Ministry of Health, Gedaref State, Sudan (Unpublished data). The four villages were Abukishma (14.05328°N, 035.12329°E), Algadamblia Tirfa (14.01715°N, 035.00059°E) Algadamblia Aljabal (14.02141°N, 035.00466°E) and Abualnaja (13.97850°N, 035.30479°E) (Fig. 1).

#### Data collection tools and procedures

Participants with chronic diseases such as Tuberculosis and HIV, and those refused to provide informed consent were excluded from the study. A total of 95 participants were recruited using a simple random selection method; 52 from Abukishma, 15 Algadamblia Tirfa, 25 Abualnaja and 3 from Algadamblia Aljabal. All participants’ demographical data including age, gender, occupation, marital status, and education, as well as household information concerning the type of the house, sleeping habitat and insect control strategies used, were collected.

#### Collection and processing of blood samples

Three milliliters of venous blood was collected from each participant from the antecubital vein by a phlebotomist. Blood samples were slowly poured into Potassium Ethylene diamine tetraacetic acid (K3-EDTA) containers to prevent coagulation. Each blood sample was gently and adequately mixed by inverting the container to avoid hemolysis, clotting, or platelet aggregation [17]. The samples were centrifuged at 1008*g* for 5 min to separate plasma and buffy coat. Plasma and buffy coat were collected into separate tubes and stored at − 20 °C for serological and molecular analysis.

#### Serological analysis

The serological analysis was performed using the direct agglutination test (DAT). DAT was performed in the Biomedical Research Laboratories of Ahfad University for Women-Sudan following the protocol described by El Harith et al. [18]. The antigen was prepared from the local endemic strain (MHOM/68/1-S). In brief, plasma diluent was made up of NaCl and gelatin of 75% (v/v), warmed at 60 °C for 15 min. 50 μl of plasma diluent was dispensed into the wells of the V-shaped microtiter plates. 1 μl of each patient plasma were added. A serial dilution was done. 50 μl of the prepared antigen was added to all the wells and incubated for 18 h at 25 °C. Results were interpreted via visualizing agglutination. A serial of titers was recorded. A titer of ≤ 1:800 was considered negative, a titer of 1:1600 was considered as borderline and a titer of ≥ 1:3200 was considered positive for VL according to El Harith et al. [19].

#### Molecular analysis

DNA was extracted from the buffy coats using the guanidine hydrochloride extraction method described previously [20]. The primers 18S-LEISH forward: 5′GCTGTGCAGGTTTGTTCCTG′3 and 18S-LEISH reverse: 5′GGACGCACTAAACCCCTCAA′3, were used to amplify a band of 357 bp within the 18S rRNA gene of *L. donovani*. PCR was performed in 25 µl reaction volume using i-Taq PreMix Kit (iNtRON, South Korea) according to the manufacturer’s instructions. PCR was performed on a thermocycler (SensoQuest, Germany). Cycling conditions were: initial denaturation at 95 °C for 5 min, 35 cycles of denaturation for 1 min at 94 °C, annealing for 1 min at 58.4 °C and elongation for 2 min at 68 °C. Also, a final elongation for 15 min at 72 °C. Distilled water was used as a negative control, and known *L. donovani* DNA was used as a positive control in each run. After amplification, the products were separated on a 2% agarose gel in TBE buffer and stained with 3 μl ethidium bromide. 5 µl of each PCR product were loaded on the gel and subjected to a current of 80 V for 90 min. The PCR products were visualized using ultraviolet trans-illuminator. The molecular weights of the amplicons were estimated with a standard 100 bp DNA ladder.

#### Collection and morphological identification of sandflies

Based on the nocturnal periodicity of the sandflies [21], sandflies collection was carried out simultaneously inside and outside the houses of the recruited participants between 18.00 and 06.00 h on four subsequent nights. 25 Sticky oil traps and 15 CDC miniature light traps were situated at 30 cm above ground level inside and outside the houses. Besides, the pyrethroid spray catch (PSC) method using Flytex aerosol was used between 06:00 and 9:00 h to collect sandflies inside the rooms of the participants. Collected sandflies were preserved in RNAlater solution (iNtRON, South Korea). The identification process was done under a binocular microscope at 40× lens following the identification keys published previously [22, 23].

#### Statistical analysis

Statistical analysis was performed using the statistical package for social sciences (SPSS. Version 16). Demographical data like age, gender, as well as household information, and the use of bed nets, insect bites frequency were analyzed. Variables considered as risk factors, including vegetation in/around the house, breeding animals, insect bites, and insect control methods were analyzed using the Chi square test.

### Results

#### Participants’ demographic data

Majority of the study participants were females; 56 (58.9%). Males constituted 39 (41.1%). Frequency of participants aged > 40 years was 54.7%, and those ≤ 40 years were 45.3%. The majority of participants 51 (53.7%) were farmers. Almost two-thirds of the participants have thatched houses with windows (55.8%). Participants who do not visit any forests were 67.4%. Participants who have vegetations inside the house were higher than those who have vegetations around the house; 78 (82.1%) and 17 (17.9%) respectively. Animal breeding inside the house was frequent than breeding animals outside the house; 85 (89.5%) and 10 (10.5%), respectively. Complaints of nocturnal and crepuscular insect bites were reported by 67 (70.5%) participants and the most frequent insects biting control method was using bed nets 58 (61.1%) (Table 1).

**Table 1:** Demographic and household information of the study participants

	Study villages				Total	P value
	Abukishma	Algadamblia Aljabal	Algadamblia Tirfa	Abualnaja		
Gender						
Male	18 (46.2%)	1 (2.6%)	12 (30.8%)	8 (20.5%)	39 (41.1%)	0.010
Female	34 (60.7%)	2 (3.6%)	3 (5.4%)	17 (30.4%)	56 (58.9%)	
Participants age						
≤ 40 years	19 (44.2%)	3 (7.0%)	9 (20.9%)	12 (27.9%)	43 (45.3%)	0.318
> 40 years	33 (63.5%)	0 (0.0%)	6 (11.5%)	13 (25.0%)	52 (54.7%)	
Marital status						
Married	46 (61.3%)	1 (1.3%)	10 (13.3%)	18 (24.1%)	75 (78.9%)	0.033
Single	6 (30.0%)	2 (10.0%)	5 (25.0%)	7 (35.0%)	20 (21.1%)	
Education status						
Illiterate	7 (50.0%)	0 (0.0%)	2 (14.3%)	5 (35.7%)	14 (14.7%)	0.004
Khalwa	31 (79.5%)	1 (2.6%)	2 (5.1%)	5 (12.8%)	39 (41.1%)	
Educated	14 (50.0%)	2 (7.1%)	11 (17.9%)	15 (25.0%)	42 (44.2%)	
Occupation						
Farmer	34 (66.7%)	1 (2.0%)	7 (13.7%)	9 (17.6%)	51 (53.7%)	0.230
Student	7 (41.2%)	2 (11.8%)	4 (23.5%)	4 (23.5%)	17 (17.9%)	
Teacher	3 (42.9%)	0 (0.0%)	2 (28.6%)	2 (28.6%)	7 (7.4%)	
Housewife	5 (45.5%)	0 (0.0%)	1 (9.1%)	5 (45.5%)	11 (11.6%)	
Driver	3 (33.3%)	0 (0.0%)	1 (11.1%)	5 (55.6%)	9 (9.5%)	
Household type						
Thatched with windows	21 (39.6%)	3 (5.7%)	13 (24.5%)	16 (30.2%)	53 (55.8%)	0.007
Thatched without windows	31 (75.6%)	0 (0.0%)	1 (2.4%)	9 (22.0%)	41 (43.2%)	
Bricked house	0 (0.0%)	0 (0.0%)	1 (100%)	0 (0.0%)	1 (1.1%)	
Forests visiting						
Yes	23 (74.2%)	0 (0.0%)	1 (3.2%)	7 (22.6%)	31 (32.6%)	0.045
No	29 (45.3%)	3 (4.7%)	14 (21.9%)	18 (28.1%)	64 (67.4%)	
Vegetations						
Inside the house	41 (52.6%)	3 (3.8%)	13 (16.7%)	21 (26.9%)	78 (82.1%)	0.870
Around the house	11 (64.7%)	0 (0.0%)	2 (11.8%)	4 (23.5%)	17 (17.9%)	
Animal breeding						
Inside the house	44 (51.8%)	3 (3.5%)	14 (16.5%)	24 (28.2%)	85 (89.5%)	0.358
Outside the house	8 (80.0%)	0 (0.0%)	1 (10.0%)	1 (10.0%)	10 (10.5%)	
Insects biting						
Crepuscular	13 (100%)	0 (0.0%)	0 (0.0%)	0 (0.0%)	13 (13.7%)	0.001
Nocturnal	5 (33.3%)	0 (0.0%)	10 (66.7%)	0 (0.0%)	15 (15.8%)	
Crepuscular and nocturnal	34 (5.7%)	3 (4.5%)	5 (7.5%)	25 (37.3%)	67 (70.5%)	
Insects control						
Aerosols	0 (0.0%)	0 (0.0%)	0 (0.0%)	3 (100%)	3 (3.2%)	0.001
Aerosols and bed nets	0 (0.0%)	0 (0.0%)	0 (0.0%)	3 (100%)	3 (3.2%)	
Bed nets	35 (60.3%)	3 (5.2%)	13 (22.4%)	7 (12.1%)	58 (61.1%)	
Repellants	0 (0.0%)	0 (0.0%)	0 (0.0%)	1 (100%)	1 (1.1%)	
Smoke	2 (16.7%)	0 (0.0%)	1 (8.3%)	9 (75.0%)	12 (12.6%)	
Smoke and bed nets	2 (100%)	0 (0.0%)	0 (0.0%)	0 (0.0%)	2 (2.1%)	
No control method	13 (76.5%)	0 (0.0%)	1 (5.9%)	3 (17.6%)	17 (17.9%)	

#### Prevalence of *Leishmania donovani* complex antigen

An overall prevalence of 5.2% VL infections were detected among the study participants. Four participants had a titer of > 1:6400 and one had a titer of 1:3200. Most of the participants did not have VL infection previously (95.8%), whereas 4/5 of those with a positive DAT titer have been previously infected with VL (Table 2).

**Table 2:** the distribution of DAT and PCR tests for the detection of VL positive participants and the different methods used for the collection of sandflies in the study villages

	Villages				Total	P value
	Abukishma	Algadamblia Aljabal	Algadamblia Tirfa	Abualnaja		
DAT and PCR results						
No. positive for DAT^a^ (%)	2 (40.0%)	1 (20.0%)	2 (40.0%)	0 (0.0%)	5 (5.3%)	0.714
No. positive for PCR (%)	19 (54.3%)	2 (5.7%)	7 (20.0%)	7 (20.0%)	35 (36.8%)	
No. examined (%)	52 (54.7%)	3 (3.2%)	15 (15.8%)	25 (26.3%)	95 (100%)	
Distribution of sandflies						
No. (%) caught using PSC	23 (42.6%)	14 (25.9%)	7 (13.0%)	10 (18.5%)	54 (24.5%)	0.006
No. (%) caught using sticky traps	51 (54.8%)	9 (9.7%)	2 (2.2%)	31 (33.3%)	93 (42.3%)	
No. (%) caught using CDC light traps	31 (42.5%)	13 (17.8%)	12 (16.4%)	17 (23.3%)	73 (33.2%)	
No. (%) of flies caught	105 (47.7%)	36 (16.4%)	21 (9.5%)	58 (26.4%)	220 (100%)	

^a^Titers were 1:100, 1:200, 1:400, 1:800, 1:1600, 1:3200, 1:6400, 1:12,800, 1:25,600, 1:51,200, and 1:102,400. A titer of ≤ 1:800 was considered negative, a titer of 1:1600 was considered as borderline, and a titer of ≥ 1:3200 was considered positive

#### PCR results

*Leishmania donovani* DNA was found in 35 out of 95 samples (36.8%). Five of the samples were found to be positive using the DAT test were also positive by PCR. The distribution of VL infected participants detected by PCR according to the location of sample collection is also illustrated in Table 2. Representative samples of PCR amplification of the 18S rRNA gene of *L. donovani* is shown in Additional file 1.

#### Abundance and distribution of sandflies vectors

A total of 220 sandflies were collected. All the collected sandflies were identified as *P. papatasi*, and none of the flies were identified as *P. orientalis*. Of the 220 sandflies, a total of 93 were collected using sticky oil traps, 73 collected using CDC light traps and 54 were collected by PSC method. 105 sandflies were collected from Abukishma, 58 from Abualnaja, 36 from Algadamblia Aljabal, and 21 from Algadamblia Tirfa. The difference of the sandflies abundance between the different villages had a statistical significance, *P* value 0.006 (see also Table 2). Additional file 2 shows the identification keys used for the identification of the sandflies.

#### Association between VL, demographic and environmental variables

The analysis of risk factors associated with VL infection did not reveal a statistically significant association. Regarding the positive participants, all positive participants, 35 (100%) were resident for more than 5 years, P-value 0.567, while those who live in a thatched house with windows were 57.1%, P-value 0.762. Also, positive participants who planted *Balanites/Acacia* trees were 21 (60.1%), and those who bred domestic animals were 32 (91.4%), P-values 0.361 and 0.211, respectively. Of the 35 (100%) positive participants who have insects biting compliance, only 20 (57.1%) were using bed nets as a control method, P-value 0.218 (Additional file 3).

### Discussion

In this study, the detected PCR-positive individuals for whom clinical VL did not develop are considered to be asymptomatic, and they have no history of previous VL infections or treatment, with no clinical signs or symptoms. However, these asymptomatic cases might act as reservoirs for the *Leishmania* parasite [24] or sustain the parasite transmission in those non-endemic regions [25]. Although the actual estimate of asymptomatic cases and their prospective role in the transmission of *L. donovani* in endemic areas is difficult to assess [26], this may escalate the challenge for the disease control [27]. VL is challenging to diagnose despite the accessibility of numerous diagnostic techniques. A single diagnostic method is not satisfactory to detect all positive VL infections, and the results obtained through multiple diagnostic methods vary from one region to another. The variable diagnostic performance of these methods in VL endemic regions is reflective of the origin of the test-antigen [27–29].

In this study, although higher number of infected persons lived in thatched houses having *Balanitis/Acacia* trees in/around the house, which is in agreement with other studies that the low financial status and mud/thatched houses or splintered houses’ walls as risk factors for VL [26, 30–34], however, there was no significant association found in this study. Also, most cases of VL were distributed among people having domesticated animals, which is in agreement with other studies, although there was no significant association were found [16, 35–42]. Also, herding of animals in/around the house was considered to have a protective role, where the domesticated animals were acting as a barrier from sandfly bites because the sandflies shifted to feed on the animals [31, 43].

Factors associated with VL may change over time, resulting in conflicting reports of their effect, such as the use of bed nets or implementation of insecticides. Insecticides may not eradicate sandflies since sandflies can persist inside the houses. Additionally, the existence of a vector in a specified area can be misleading hence presence alone does not prove *L. donovani* transmission, which is affected by strain, behavior, seasonal activity, and density of the vector [31, 43].

The proportion of infected patients with *L. donovani*, who may act as a reservoir for in Sudan is barely documented as this requires extensive prospective epidemiological studies. However, no evidence exists demonstrates that individuals with asymptomatic *L. donovani* infection are not reservoirs. Thus, the assessment of those with asymptomatic infections by screening and up to 1-year follow-up is beneficial in VL control.

### Conclusion

Early treatment of VL-infected patients, mainly asymptomatic individuals will help to reduce disease transmission, as well as mortality.

## Limitations

- Although the results generated from this study provide insights on the status of asymptomatic VL in Sudan, the number of study participants recruited was small due to community incorporation. Therefore, a more extensive study scale is needed to give a clear situation of VL infections in Sudan. As such, the results can, therefore, be used to generalize with caution.

## Supplementary information

**Additional file 1.** Representative samples of the PCR amplification of the 18S rRNA gene of *Leishmania* parasites.

**Additional file 2.** Morphological identification of the wild-caught sandfly.

**Additional file 3.** Association between VL infection and demographic, household/environmental, behavioral factors.

## Data Availability

The datasets used and/or analyzed during the current study are available from the corresponding author on reasonable request.

## Abbreviations

DAT: direct agglutination test
HIV: human immunodeficiency virus
K3-EDTA: potassium ethylene diamine tetraacetic acid
NaCl: sodium chloride
PCR: polymerase chain reaction
PSC: pyrethroid spray catch
TBE: tris boric acid EDTA
VL: visceral leishmaniasis
