# Supplementary material for: Identifying asymptomatic Leishmania infections in non-endemic villages in Gedaref state, Sudan
Source: BMC Res Notes. 2019 Sep 11;12:566. doi: 10.1186/s13104-019-4608-2 (PMC6737656; doi:10.1186/s13104-019-4608-2)
Supplement: Supplementary file 1 — Additional file 1. Representative samples of the PCR amplification of the 18S rRNA gene of Leishmania parasites. [file 13104_2019_4608_MOESM1_ESM.docx]

**Additional File 1:**


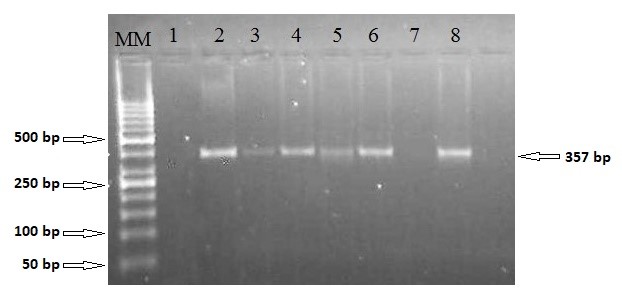


**Figure S1:** Representative samples of the PCR amplification of the 18S rRNA gene of *Leishmania* parasites. **MM:** molecular marker (100bp); **Lane 1:** negative control (Distilled water); **Lane 2:** Positive control (*L. donovani* DNA), **Lanes 3-6**, and **8:** positive individuals with VL; **Lane 7:** negative individual.
