# Supplementary material for: Identifying asymptomatic Leishmania infections in non-endemic villages in Gedaref state, Sudan
Source: BMC Res Notes. 2019 Sep 11;12:566. doi: 10.1186/s13104-019-4608-2 (PMC6737656; doi:10.1186/s13104-019-4608-2)
Supplement: Supplementary file 2 — Additional file 2. Morphological identification of the wild-caught sandfly. [file 13104_2019_4608_MOESM2_ESM.docx]

**Additional file 2:**


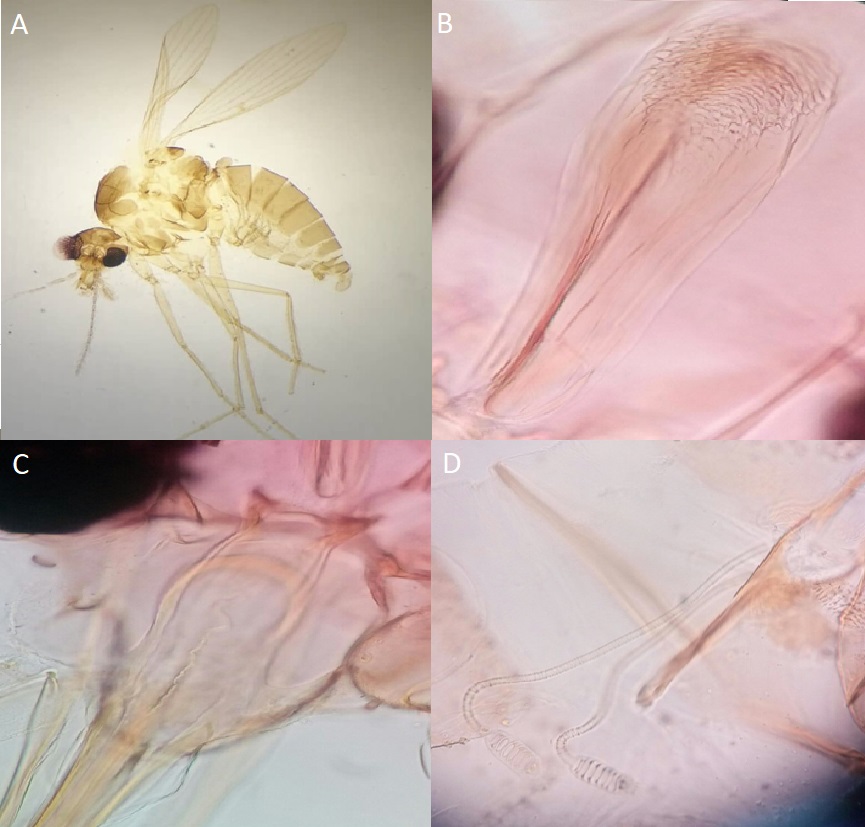


**Figure S2: Morphological identification of the wild-caught sandfly**. **A:** Adult female *P. papatasi*. The primary features used for of female sandfly identification were **B:** the pharynx in the head, **C:** the cibarium in the head and **D:** the spermatheca in the abdomen. Images show the shape of the pharynx and the cibarium in the head of the female *P. papatasi*, also showing the segmented capsule of spermatheca with striated spermathecal duct of *P. papatasi*.
