# Supplementary material for: Identifying asymptomatic Leishmania infections in non-endemic villages in Gedaref state, Sudan
Source: BMC Res Notes. 2019 Sep 11;12:566. doi: 10.1186/s13104-019-4608-2 (PMC6737656; doi:10.1186/s13104-019-4608-2)
Supplement: Supplementary file 3 — Additional file 3. Association between VL infection and demographic, household/environmental, behavioral factors. [file 13104_2019_4608_MOESM3_ESM.docx]

**Additional file 3:**

**Table S1:** Association between VL infection and demographic, household/environmental, behavioral factors.

| **Factor** | **Category** | **PCR Positive (35)** | | **P value** |
| --- | --- | --- | --- | --- |
|  |  | **Number** | **%** |  |
| Gender | Male | 14 | 40.0 | 0.542 |
|  | Female | 21 | 60.0 |  |
| Participants age | < 40 years | 16 | 45.7 | 0.739 |
|  | ≥ 40 years | 19 | 54.3 |  |
| Education | Illiterate | 8 | 22.9 | 0.312 |
|  | Khalwa | 14 | 40.0 |  |
|  | Educated | 13 | 37.1 |  |
| Marital status | Single | 7 | 20.0 | 0.564 |
|  | Married | 28 | 80.0 |  |
| No. of family members | 1 – 5 | 13 | 37.1 | 0.133 |
|  | More than 6 | 22 | 62.9 |  |
| Occupation | Farmer | 19 | 54.3 | 0.257 |
|  | Student | 5 | 14.3 |  |
|  | Teacher | 5 | 14.3 |  |
|  | Housewife | 3 | 8.6 |  |
|  | Driver | 3 | 8.6 |  |
| Residence | More than 5 years | 35 | 100 | 0.567 |
| Forest visits | No visits | 24 | 68.6 | 0.344 |
|  | Visits | 11 | 31.7 |  |
| Sleeping habit | On bed | 35 | 100 | 0.964 |
| Prevention methods | Bed nets | 20 | 57.1 | 0.218 |
|  | Others | 7 | 20.0 |  |
|  | No prevention method | 8 | 22.9 |  |
| Household type | Thatched with windows | 20 | 57.1 | 0.762 |
|  | Thatched without windows | 14 | 40.0 |  |
|  | Bricked house | 1 | 2.9 |  |
| Vegetations in/around the house | *Balanites/Acacia* trees | 21 | 60.1 | 0.361 |
|  | Others | 9 | 25.9 |  |
|  | No trees | 5 | 12.8 |  |
| Animals breeding | Cow, sheep, goat, donkey, dog, cat, chicken | 32 | 91.4 | 0.211 |
|  | No animals | 3 | 8.6 |  |
| Insect bites at night | Yes | 35 | 100 | 0.140 |
